# Data for the level of women׳s self-esteem and couples’ sexual satisfaction before and after mammoplasty

**DOI:** 10.1016/j.dib.2018.07.002

**Published:** 2018-07-10

**Authors:** Mohammad Sahebalzamani, Khadijeh Chale Chale, Hojjatollah Farahani

**Affiliations:** a Department of Management, Tehran Medical Sciences Branch Islamic Azad University, Tehran, Iran; b Kermanshah University of Medical Sciences, Kermanshah, Iran; c Department of Psychology, Tehran Medical Sciences branch Islamic Azad University, Tehran, Iran; d Psychometrics, Australia

**Keywords:** Women, Mammaplasty, Sexual satisfaction, Self–esteem

## Abstract

Nowadays, one of the existing problems in the societies is the increase in the rate of plastic surgeries such as mammaplasty, especially among the women in Iran. The present study was conducted on the positive and negative effects of this surgery on Iranian women׳s self-esteem and couples’ sexual satisfaction before and after mammaplasty. The analysis is based on data of a pretest -posttest design with only one group conducted on 100 couples. Three questionnaires of demographic characteristics, Rosenberg׳s self-esteem, and women׳s and men׳s sexual satisfaction were adopted. The questionnaires were completed before surgery and two months after by the couples. Among 100 studied participants, mean self-esteem before and after mammaplasty were 18.77 and 17.96 respectively. Mean women׳s sexual satisfaction before and after surgery were 30.80 and 39.80 respectively. In conclusion, mammaplasty is effective on increase of women׳s sexual satisfaction, but it has no effect on increase of their self-esteem and their husbands’ sexual satisfaction.

**Specifications Table**

**Table:** 

Subject area	Psychometrics
More specific subject area	Clinical psychometrics
Type of data	Tables
How data was acquired	The analysis is based on data of a pretest-posttest design with only one group conducted on 100 couples. Three questionnaires of demographic characteristics, Rosenberg׳s self-esteem, and women׳s and men׳s sexual satisfaction were adopted.
Data format	Analyzed
Experimental factors	The reliability of the questionnaires was analyzed using Cronbach׳s alpha.
Experimental features	The questionnaires were completed before surgery and two months after by the couples.
Data source location	Kermanshah, Kermanshah province, Iran.
Data accessibility	Data are included in this article

**Value of the data**

• Mammaplasty is a type of plastic surgery that women seek for improvement of their own and their husbands’ sexual satisfaction. There are limited data on the effect of mammoplasty on the self-esteem of women and the relationship between women and men [1], [2], [3].
• This data covers the inadequacy of information concerning sexual issues and existence of wrong beliefs and attitudes toward this subject and its modification interventional methods.
• The data in this article reveals that the self-esteem of women before and after mammaplasty has not changed, but it improved the sexual satisfaction of couples.

## 1 Data

Out of 100 investigated couples, 3% of the women were under 20 years of age, 17% between 20–30 years, 49% between 30–40 years and 31% over age of 40. About 57% of their spouses were employees and 43% were self-employed. About 19% of the couples had no children, 19% had one child, 55% had two children and 7% had more than two children. About 66% underwent a breast reduction surgery, 19% breast augmentation and 15% underwent mastopexy. About 19% predicted to achieve beauty less than 30%, 41% between 30–50%, 34% between 50–70% and 6% predicted to achieve over 70% of beauty. About 16% of the women had history of a plastic surgery while 84% did not. Meanwhile, 5% of the women had a history of plastic surgery in their immediate family members while 95% did not. About 53% of the women had a history of surgery in their friends and relatives while 47% mentioned no history.

Table 1 presents mean (SD) of couples’ self-esteem and sexual satisfaction before and after mammaplasty surgery. Mean score of self-esteem was 18.77 (moderate) before surgery in 100 participants, and it remained steady (17.96) after the surgery (moderate).

**Table 1:** Mean (SD) of couples’ self-esteem and sexual satisfaction before and after mammaplasty surgery.

**Variable**	**Number**	**Mean**	**SD**
Women׳s self-esteem before surgery	100	18.77	4.50
Women׳s self-esteem after surgery	100	17.96	3.39
Women׳s sexual satisfaction before surgery	100	30.84	6.32
Women׳s sexual satisfaction before surgery	100	39.80	8.73

Table 2 shows the mean (SD) of couples’ self-esteem and sexual satisfaction before and after mammaplasty surgery. Women׳s sexual satisfaction was increased after mammoplasty; however, their satisfaction did not change.

**Table 2:** Couples’ sexual satisfaction and women׳s self-esteem before and after mammaplasty surgery.

	**Before surgery**		**After surgery**	
	**Mean**	**SD**	**Mean**	**SD**
Women׳s self esteem	18.77	4.50	17.96	3.39
Women׳s sexual satisfaction	30.84	6.32	39.80	8.73
Men׳s sexual satisfaction	61.98	10.14	99.73	9.98

## 2 Experimental design, materials and methods

A pretest -posttest design with only one group was contacted among 100 clients referring to 5 hospitals affiliated to Kermanshah University of Medical Sciences for a mammaplasty surgery in 2013. 100 couples were selected by purposive sampling, when referring to the hospitals affiliated to Kermanshah University of Medical Sciences. Participation inclusion criteria included being married with at least one year of marriage length, living with their spouse, having reading and writing literacy, being a candidate for mammaplasty surgery, and referring to hospitals in Kermanshah province. The couples with the history of a diagnosed mental disease, taking psychotropic medications and those with unpleasant postoperative complications were left out of study.

The first contained participants’ demographic characteristics including women׳s and their spouses’ level of education, women׳s and their spouses’ occupational status, number of children, type of surgery, prediction of beauty percentage, history of any previous plastic surgeries among the participants and their immediate family members, relatives and friends. To establish the validity of demographic questionnaire, content validity was used through taking indications of 10 academic members.

The second questionnaire was couples’ sexual satisfaction questionnaire, which is a self-assessment tool to measure the level of marital satisfaction in sexual issues and includes items on sexual satisfaction, sexual shyness, sexual trenchancy, sexual anxiety, sexual health, and fear and sexual compulsion in couples’ marital relationship. This questionnaire was designed in two separate forms for women and men. The men׳s version contains 28 items which are scored in a five point Likert scale (absolutely agree, agree, no idea, disagree, absolutely disagree) ranging between 0–4 points. Each subject is scored between 0–108. Scores 0–27 show low sexual satisfaction; 28–54 show moderate, 55–81 show good sexual satisfaction,and scores 82–108 show high sexual satisfaction. The women׳s version of this questionnaire contains 17 items, scored in a five- point Likert scale (absolutely agree, agree, no idea, disagree, absolutely disagree), ranging between 0–4 points. Participants’ total score ranges 0–68. Scores 0–17 show low sexual satisfaction; 18–34 show moderate; 35–51 show high and scores 52–68 show very high sexual satisfaction. To check reliability of the women׳s and men׳s sexual satisfaction questionnaires, Cronbach׳s alpha method was adopted. In the present study, reliability of this tool was calculated 0.84 through Cronbach׳s alpha in a 20- male subject sample in a pilot study and 0.83 in a 20-female sample.

The third questionnaire was Rosenberg Body-esteem Scale containing 10 general items (five items with negative and five items with positive sentences). Each item is scored with a four- point Likert scale (absolutely agree, agree, disagree, absolutely disagree) ranging between 0–3 with total scores of 0–40. Scores 0–10 show very low self-esteem; 10–20 show moderate; 20–30 show high and score, 30–40 show very high self-esteem. In the present study, internal consistency of the tool was calculated 0.82 through Cronbach׳s alpha of 0.82 on 20 participants.

After getting permission from research chancellery of Kermanshah University of Medical Sciences and attaining the consent from the participants, the questionnaires were administered among the participants in an appropriate place, and they were explained about the goals of study and the way to fill the questionnaires. The participants were investigated before the surgery and two months after, and finally, the obtained data were analyzed.

All obtained data in the present study were confidential and the participants were assured about their anonymous questionnaires. Collected data were summarized using descriptive statistics namely absolute (n) and relative (%) frequencies for categorical variables and mean and standard deviation (SD) to study the level of women׳s self-esteem and couples’ sexual satisfaction before and after mammaplasty,
